# The Diversity of Arachnid Assemblages on the Endemic Tree *Zelkova abelicea* (Ulmaceae): An Evaluation of Fragmentation and Connectivity in Crete (Greece)

**DOI:** 10.3390/insects15100788

**Published:** 2024-10-10

**Authors:** Dariusz J. Gwiazdowicz, Laurence Fazan, Giulio Gardini, Dany Ghosn, Sławomir Kaczmarek, Alireza Nemati, Ilektra Remoundou, Tomasz Rutkowski, Piotr Skubała, Bogna Zawieja, Gregor Kozlowski

**Affiliations:** 1 Faculty of Forestry and Wood Technology, Poznań University of Life Sciences, Wojska Polskiego 71c, 60-625 Poznań, Poland; 2 Department of Biology and Botanic Garden, University of Fribourg, Chemin du Musée 10, 1700 Fribourg, Switzerland; laurence.fazan@unifr.ch (L.F.); gregor.kozlowski@unifr.ch (G.K.); 3 Via Monte Corno 12/1, 16166 Genova, Italy; giuliogardini@libero.it; 4 Department of Geoinformation in Environmental Management—Alsyllio Agrokepiou, CIHEAM Mediterranean Agronomic Institute of Chania, 73100 Chania, Greece; dghosn@maich.gr (D.G.); hlektra@maich.gr (I.R.); 5 Department of Evolutionary Biology, Faculty of Biological Sciences, Kazimierz Wielki University, Ossolińskich Av. 12, 85-094 Bydgoszcz, Poland; slawkacz@ukw.edu.pl; 6 Plant Protection Department, Agricultural College, Shahrekord University, Shahrekord 8818634141, Iran; nemati.alireza@sku.ac.ir; 7 Natural History Collections, Faculty of Biology, Adam Mickiewicz University, Uniwersytetu Poznańskiego 6, 61-614 Poznań, Poland; pardosa@gazeta.pl; 8 Faculty of Natural Sciences, University of Silesia in Katowice, Bankowa 9, 40-007 Katowice, Poland; piotr.skubala@us.edu.pl; 9 Department of Mathematical and Statistical Methods, Poznan University of Life Sciences, Wojska Polskiego 28, 60-637 Poznań, Poland; bogna13@up.poznan.pl; 10 Natural History Museum Fribourg, Chemin du Musée 6, 1700 Fribourg, Switzerland; 11 Shanghai Chenshan Plant Science Research Center, Plant Systematics and Evolutionary Biology, Chinese Academy of Sciences, Shanghai 201602, China

**Keywords:** Acari, Araneae, Pseudoscorpiones, biodiversity, ecology of arthropods, zoogeography

## Abstract

**Simple Summary:**

*Zelkova abelicea* is an endemic tree growing only on eight mountain stands on the Greek island of Crete. The aim of this study was to determine the structure of the assemblages and analyze the diversity of the arachnid assemblages (spiders, pseudoscorpions, mites) living on these trees. Samples for the analyses were collected from tree trunks, oftentimes covered by bryophytes or lichens. In the examined material, 85 taxa were recorded. The most numerous represented group was mites (1134 specimens, 69 species), and the highest numbers of specimens were in the order Oribatida, namely *Zygoribatula exilis* (284 specimens) and *Eremaeus tuberosus* (210). Additionally, in order Mesostigmata, *Hypoaspisella* sp. was found, which is probably a species new to science. Among the eight sampled localities, Gerakari (646 specimens, 50 species) and Omalos (409, 43) had by far the richest assemblages. Our statistical analyses confirmed the highly diverse character of the arachnid assemblages at the individual sites, which is a consequence not only of the varied numbers of arachnids found, but also of the presence of very rare species, such as *Androlaelaps shealsi*, *Cosmolaelaps lutegiensis* or *Hoploseius oblongus* in the order Mesostigmata. These results highlight the high species diversity of arachnids found on *Z. abelicea* but also suggest the lack of connectivity between the isolated and fragmented forest stands on Crete.

**Abstract:**

*Zelkova abelicea* is an endemic tree growing only on eight mountain stands on the Greek island of Crete. The aim of this study was to determine the structure of the assemblages and analyze the diversity of the arachnid assemblages living on *Zelkova abelicea*, an endemic tree species in Crete. Material for the analyses was collected from tree trunks, oftentimes covered by bryophytes or lichens. In the examined material, 85 taxa were recorded. The most numerous groups represented in the analyzed material were Acari, including representatives of the orders Mesostigmata (78 ind. of 18 spp.) and Oribatida (1056 ind. of 51 spp.). In the order Mesostigmata the species represented by the highest numbers of specimens were *Onchodellus karawaiewi* (15 individuals) and *Hypoaspisella* sp. (13), which is probably a species new to science. In turn, representatives of the order Oribatida were much more numerous, with *Zygoribatula exilis* (284) and *Eremaeus tuberosus* (210) being identified in the largest numbers. Among the eight sampled localities, Gerakari (646 ind. and 50 spp.) and Omalos (409 ind. and 43 spp., respectively) had by far the richest assemblages. Statistical analyses confirmed the highly diverse character of the arachnid assemblages at the individual sites, which is a consequence not only of the varied numbers of arachnids found, but also of the presence of very rare species, such as *Androlaelaps shealsi*, *Cosmolaelaps lutegiensis* or *Hoploseius oblongus*. These results highlight the high species diversity of the arachnids found on *Z. abelicea* but also suggest the lack of connectivity between the isolated and fragmented forest stands on Crete.

## 1. Introduction

Arachnids play an important role in ecosystems by contributing to the local biodiversity but also because they are important components of the food web [1]. Although taxonomic work on specific groups within the arachnids has been carried out for Crete (e.g., Araneae [2], Acari [3,4,5,6,7] or Pseudoscorpiones [8]), faunistic studies remain rare, but highlight a high local diversity [9]. However, there remains still much to be discovered about community composition and diversity, notably regarding the arachnid fauna associated with specific plant or tree species growing in the area.

Relicts (from Latin *relictus*—left behind, relinquished; past participle of *relinquere*—to relinquish, to leave behind) are taxa that remain from a previously geographically and/or phylogenetically larger group [10]. Examples of relict tree species belong to such genera as *Aesculus*, *Juglans*, *Laurus*, *Liquidambar*, *Parrotia*, *Pterocarya*, *Rhododendron* or *Zelkova* [11]. Many species from these genera are rare and/or threatened globally and should deserve protection [11,12]. This need is also related to the fact that they create unique microhabitats, which support a rich biodiversity, oftentimes comprising rare or relict taxa [13,14,15,16,17,18,19].

The genus *Zelkova* is a relict of the Arctotertiary geoflora [20], and its species were important forest components in the Northern Hemisphere during the Paleogene. Six extant species of the genus occur with a disjunct distribution. Three species are found in eastern Asia (*Z. serrata* (Thunb.) Makino, *Z. schneideriana* Hand.-Mazz. and *Z. sinica* Schneid). One species grows in the Middle East and Transcaucasia *Z. carpinifolia* (Pall.) Koch. *Zelkova sicula* Di Pasq. et al. and *Z. abelicea* (Lam.) Boiss. are endemic to the Mediterranean islands of Sicily and Crete, respectively. Five of the six *Zelkova* species are endangered and therefore protected in several countries. The rarest and most endangered species are the two above-mentioned Mediterranean species, which is a result of intensive forest management and industrialized agriculture, as well as the loss of habitats, droughts and water shortages [11].

*Zelkova abelicea* grows in several localities in small and fragmented populations in the mountainous regions of Crete, above 800 m a.s.l. [12,21]. The majority of individuals have a dwarfed shrubby life-form, with extremely slow growth rates [21] resulting from over browsing by goats. In contrast, tree individuals with the life-form of a tree are far less common, reaching 15–20 m in height. The biodiversity associated with *Z. abelicea* has been poorly studied and no thorough research has been conducted to date on invertebrates colonizing *Z. abelicea*. Only two Phytoseiidae and one Parasitidae mite (Acari) have been recorded [5,7], as well as one Hymenoptera species [22]. Gwiazdowicz et al. [19] showed over 30 species of springtails (Collembola), including species new to science.

The aim of this study was to determine the community structure and the species diversity of arachnid assemblages from *Z. abelicea* specimens growing in several localities throughout Crete. We hypothesized that the arachnid assemblages on *Z. abelicea* will be diverse and different at each locality due to the isolation and fragmented nature of *Z. abelicea* stands, with no evident connections between the stands (also due to the particular geomorphology of Crete).

## 2. Methods

### 2.1. Field Studies

**Figure 1:**
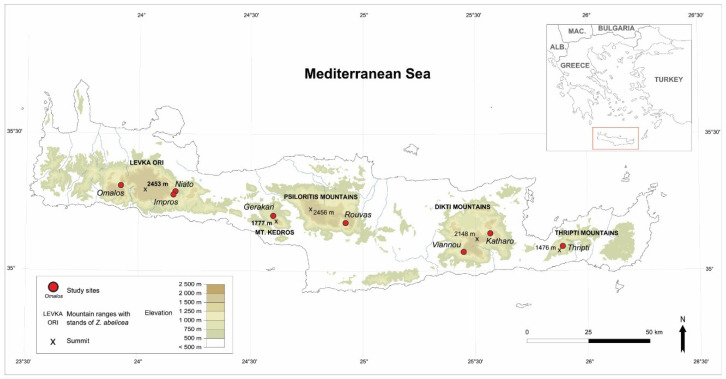
Sampled localities (red dots) in Crete (Greece) with *Zelkova abelicea* trees.

The arachnids were collected at eight study sites distributed over the entire range of *Z. abelicea* in Crete (Figure 1).

1. Omalos, Levka Ori (Latitude 35.31901; Longitude 23.91871), Altitude—1160 m a.s.l., topography—slope, microhabitat—bark of arborescent trees, date—21 May 2019, Coll. D. Ghosn;
2. Niato, Levka Ori (35.287527; 24.145503), 1215 m a.s.l., doline, branches of dwarfed individuals, 21 May 2019, Coll. D. Ghosn;
3. Impros, Levka Ori (35.270546; 24.15315), 1175 m a.s.l., slope, bark of arborescent trees, 21 May 2019, Coll. D. Ghosn;
4. Gerakari, Mt. Kedros (35.194829; 24.606713), 1255 m a.s.l., slope, bark of arborescent trees, 11 October 2018, Coll. D.J. Gwiazdowicz;
5. Rouvas, Psiloritis Mountains, (35.164333; 24.922794), 1320 m a.s.l., slope, bark of arborescent trees, 10 October 2018, Coll. D.J. Gwiazdowicz;
6. Viannou, Dikti Mountains, (35.064291; 25.469778), 1320 m a.s.l., slope, bark of arborescent trees, 9 October 2018, Coll. D.J. Gwiazdowicz;
7. Katharo, Dikti Mountains, (35.148004; 25.567558), 1160 m a.s.l., slope, bark of arborescent trees, 9 October 2018, Coll. D.J. Gwiazdowicz;
8. Thripti, Thripti Mountains, (35.080588; 25.887408), 1150 m a.s.l., doline, branches of dwarfed individuals, 14 May 2019, Coll. D. Ghosn.

**Figure 2:**
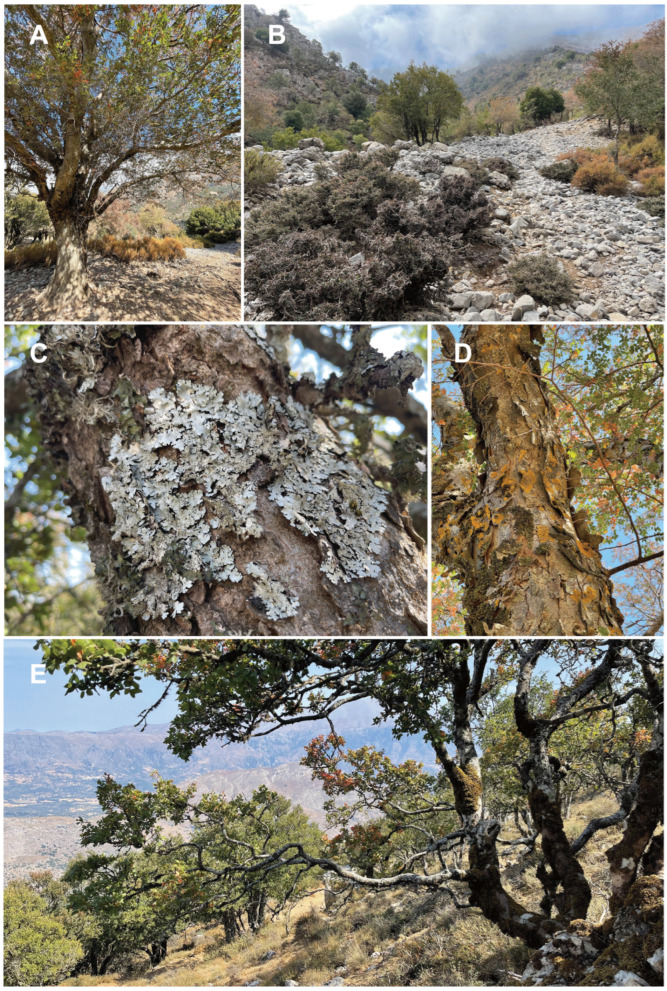
*Zelkova abelicea* trees with microhabitats for invertebrates. (**A**) Large trees (Omalos). (**B**) Dwarfed trees heavily browsed by goats (Omalos). (**C**) Bark of large tree covered by species of lichens from genus *Parmelina* (Gerakari). (**D**) Bark of large trees (Gerakari). (**E**) View of *Z. abelicea* population in Gerakari (Photos: G. Kozlowski).

At each sampling site, one sample was collected on each of five *Z. abelicea* trees growing at a distance of several to tens of meters apart. A sample of the outer trunk bark layer, oftentimes including bryophytes or lichens see Ref. [18] was cut off with a knife from well-developed arborescent trees. In the case of dwarfed trees, branches were cut off with pruning shears. This difference in treatment is due to the fact that the bark on the trunk of young or dwarf trees is smooth and thin, while on old and large trees it is thick, exfoliating and easily cut off (Figure 2). The collected material was placed in paper bags. The weight of each (fresh) sample ranged from 200 to 250 g. Study permits were granted by the Greek Ministry of Environment (no. 174101/5060 and no. 155924/1184).

### 2.2. Laboratory Procedures

The collected samples were placed into Tullgren funnels for 72 h and extracted in 96% ethanol. The extracted arthropods were classified into the following groups using a Zeiss Stemi 2000 stereoscopic microscope: Araneae, Mesostigmata, Oribatida and Pseudoscorpiones.

The collected Araneae were identified at the species level (when possible) and counted. The taxonomic keys of spiders [23,24,25] were used to identify species of Araneae under stereoscopic microscopy. For Pseudoscorpiones, some of the characteristics used for the identification can be seen using a stereomicroscope, but it is always necessary to prepare temporary microscope slides in lactic acid to examine details using an Olympus BHB compound microscope. In order to identify Mesostigmata (Acari), both semi-permanent (using lactic acid) and permanent microslides (using Hoyer’s medium) were prepared. All the mesostigmatic mites were examined using a light microscope (Zeiss Axioskop 2) and taxonomical literature [26,27,28].

The Oribatida (Acari) were identified at high magnifications (l00–1000×) under a light microscope, preferably with a phase contrast and a differential interference contrast. Prior to the examination, the cuticles were rendered transparent, and in freshly collected specimens, the internal tissue was removed using concentrated lactic acid, 60% lactic acid or lactophenol. A diluted lactic acid was used, which is considered to be more suitable with weakly sclerotized forms [29]. Another method consisted of heating the samples to 60–70 °C on a hot plate as temporary mounts in lactic acid on a cavity slide [30]. The clearing process was performed at room temperature over a course of several days, and sometimes weeks. Oribatid mites were identified to a species level by identifying their key features and original species descriptions [31,32,33].

All the investigated material is deposited in the Natural History Collections at the Adam Mickiewicz University, Poznań, Poland (Araneae), the Department of Forest Entomology and Pathology, the Poznań University of Life Sciences (Mesostigmata, Pseudoscorpiones), and the University of Silesia in Katowice, Poland (Oribatida). Microslides with the analyzed specimens have also been deposited at the CIHEAM Mediterranean Agronomic Institute of Chania in Crete and at the Natural History Museum in Athens, Greece.

### 2.3. Statistical Analyses

Descriptive statistics of arachnid assemblages were computed for individual sampling sites. The following values were computed: total species count, mean number of arachnid individuals and the standard error of the mean, as well as the minimum and maximum numbers of arachnid individuals. In addition, the following values were calculated: Simpson’s [34] diversity index (one minus sum of empirical probabilities), standard error and the minimum and maximum values of this index, while similar values were determined for Pielou’s [35] evenness index (the Shannon diversity index divided by the logarithm of the number of species). The latter cannot be computed when the number of species in a sample is zero. Therefore, computation of the indexes was conducted only of samples with arachnids. For each species, its dominance index [36] was calculated, which was understood as the share of the number of specimens of a given species in the total number of specimens (of all species found in the study) and expressed in percent. A comparison of the range and density of the distributions of specimens and species for each site is presented graphically using violin plots.

In addition, for the 10 most numerous species, their frequency (*F*%) was calculated (i.e., the percentage of samples in which the species occurred) and the relative density (*R*) (the ratio of the number of specimens of a given species to the number of all samples) along with the confidence interval for this parameter [37]. We also calculated intensity (*I*) (the ratio of the number of specimens of every species to the number of samples in which the species was found) and confidence interval for Poisson’s distributed variable [38].

Because the Acari were most frequently encountered, a non-metric multidimensional scaling (NMDS) [39] was performed using the Hellinger-transformed Bray–Curtis distance matrix [40] dissimilarity matrix for Acari species, while Wisconsin double standardization [41] was conducted conditionally in the applied procedure (if the data values are larger than common abundance class scales). In the NMDS analysis, a total of 900 random starts were made in order to obtain a global optimum.

A multilevel pattern analysis was used to indicate which Acari species prefer a similar habitat. This analysis enables the identification of species lists that were linked to specific groups of sites (or their combinations). The indicator value index was utilized for assessing the connection between a set of locations and various species. The index was computed for every species in relation to various location groups. The locations that exhibit the highest index values were selected. Subsequently, a permutation test was employed to assess the statistical significance [42]. Moreover, cluster analysis was applied using the Bray–Curtis distance matrix and the Ward method to group similar habitats colonized by Acari that were transposed by Hellinger [43] method. A heatmap was created to display the number of observed species in various locations, along with a species co-occurrence analysis showing significantly higher or lower co-occurrence frequencies than expected, as well as random frequencies. [44] Species will be considered co-occurring if they exhibit a significantly higher probability of co-occurrence than the expected frequency. Beta diversity was calculated, and cluster analysis was applied to this matrix to illustrate the similarities between locations.

All the above mentioned statistics were carried out in R (version 4.0.3) [45] using functions (diversity, metaMDS and betadiver) of the vegan package [46], as well as functions pheatmap from pheatmap package [47], function cooccur from cooccur package [48] as well as functions multipatt of the indicspecies [49] and stats packages [44] package. Violin plots were done using ggplot2 [50].

## 3. Results

### 3.1. General Information

Based on the collected material, a total of 85 taxa were recorded, of which the majority (66 spp.) were identified to the species level. Several specimens found in the larval or nymph stages were classified to the genus (13 taxa) or to the family (6 taxa) level (Appendix A).

In the order Araneae, 33 individuals from 14 taxa were recorded, but only four of them were identified to the species level. The dominance of individual species was always lower than one percent, whereas the dominance (D) of all Araneae species in the entire analyzed material amounted to 2.78%.

In the order Pseudoscorpiones, a total of 19 specimens belonging to two species were recorded, with *Beierochelifer peloponnesiacus* (Beier, 1929) being more numerous (D: 1.01%). The dominance of the order Pseudoscorpiones in the entire investigated material was 1.6%.

**Figure 3:**
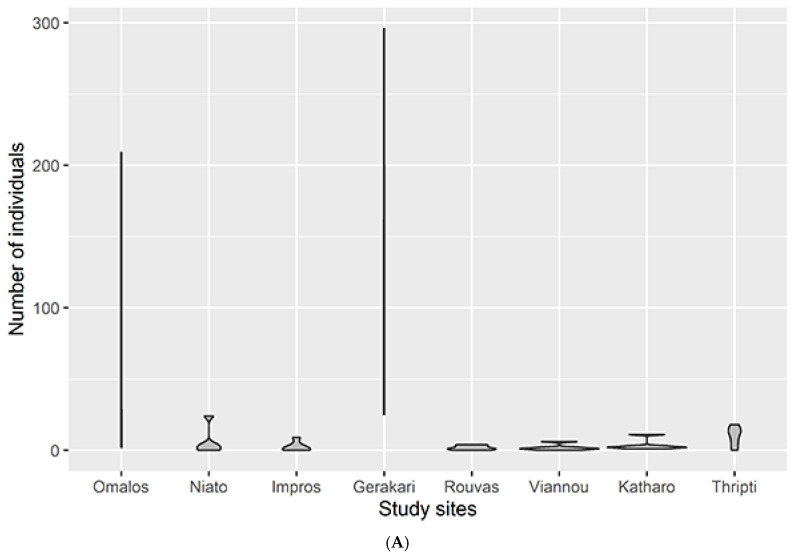

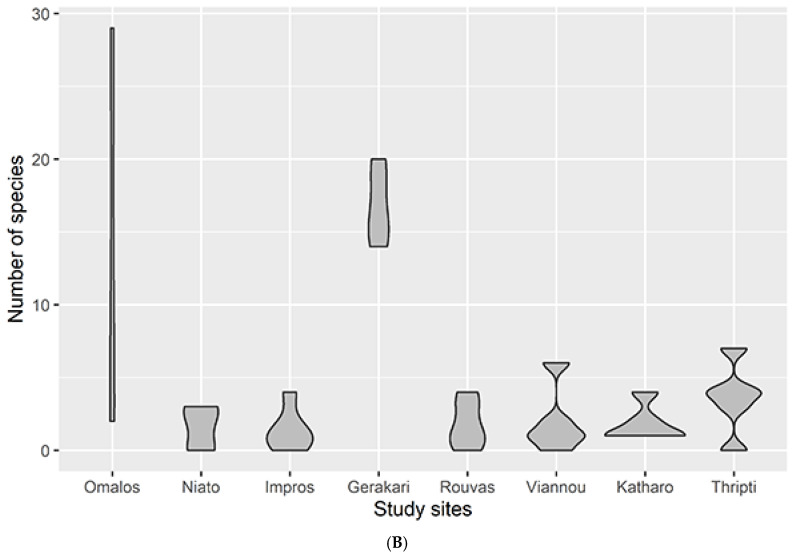
The range of arachnida specimens (**A**) and species (**B**) per study site. The width of the violin plots represents the number of individuals (**A**) or species (**B**).

In the analyzed material, Acari constituted the most numerous group, which included representatives of the orders Mesostigmata (78 ind., 18 spp., D: 6.58%) and Sarcoptiformes (suborder Oribatida; 1056 ind., 51 spp., D: 89.04%). Among the eight locations, Gerakari (646 ind. and 50 spp.) and Omalos (409 ind. and 43 spp.) were found to be the richest. All six other study sites had 50 or fewer specimens and 10 or fewer species (Table 1, Figure 3).

In Gerakari and Omalos, the number of specimens per sample varied strongly. Each sample had a different number of specimens and each species abundance occurred in only one sample, giving an almost one-dimensional violin plot (Figure 3). In the six other study sites, the sample size did not exceed 24 specimens. The shape of the graphs shows that in most samples, the number of specimens was very small (a few species). Concerning the species count, in Omalos, the samples were very diverse (min: 2, max: 29). In Gerakari, the number of species per sample was less varied but the species numbers were quite high (min: 14, max: 20). For all other sites, the species richness was less than eight and only a single individual of each species was found.

The mean biodiversity is shown per site by the Simpson index (Table 1) and was highest in Gerakari (0.81), Thripti (0.67) and Omalos (0.64), while it was smallest in Impros (0.17) and Katharo (0.19). The greatest variability (Table 1) of this parameter was found in Rouvas (the standard error of the mean was 0.21) and Viannou (0.20). In Thripti (0.88) and Omalos (0.81), the mean for Pielou’s evenness was relatively high. The lowest evenness was recorded for one sample from Niato (0.25). The scatter of this index in Niato was the greatest (max = 1.00). Generally, it may be stated that the evenness in most samples was high despite the large population size of *Zygoribatula exilis* (Nicolet, 1855) in two samples from Omalos.

Araneae were identified in samples from five sites (Omalos, Gerakari, Rouvas, Viannou and Katharo), and Pseudoscorpiones in four sites (Omalos, Impros, Gerakari and Thripti). Despite being more numerous both in species and individual counts, Mesostigmata were detected only in three sites (Gerakari, Katharo and Thripti). Acari from the order Oribatida were reported from all eight sites, although they were not present in all samples (11 samples were empty, 27.5%) (Appendix A).

### 3.2. Diversity of Acari Assemblages on Zelkova Abelicea Trees

As mentioned above, representatives of two Acari orders were identified in the collected material. In the order Mesostigmata, the most numerous species were *Onchodellus karawaiewi* (Berlese, 1920) (15 ind., D:1.26%) and *Hypoaspisella* sp. (13; 1.1%), the latter probably being a species new to science, which will be the subject of a separate taxonomic study. In turn, representatives of the order Oribatida were much more numerous, among which the species represented by the largest numbers of specimens included *Zygoribatula exilis* (Nicolet, 1855) (284; 23.95%), *Eremaeus tuberosus* Gordeeva, 1970 (210; 17.71%), *Chamobates dentotutorii* Shaldybina, 1969 (83; 7%) and *Eupelops acromios* (Hermann, 1804) (81; 6.83%) (Appendix A).

**Figure 4:**
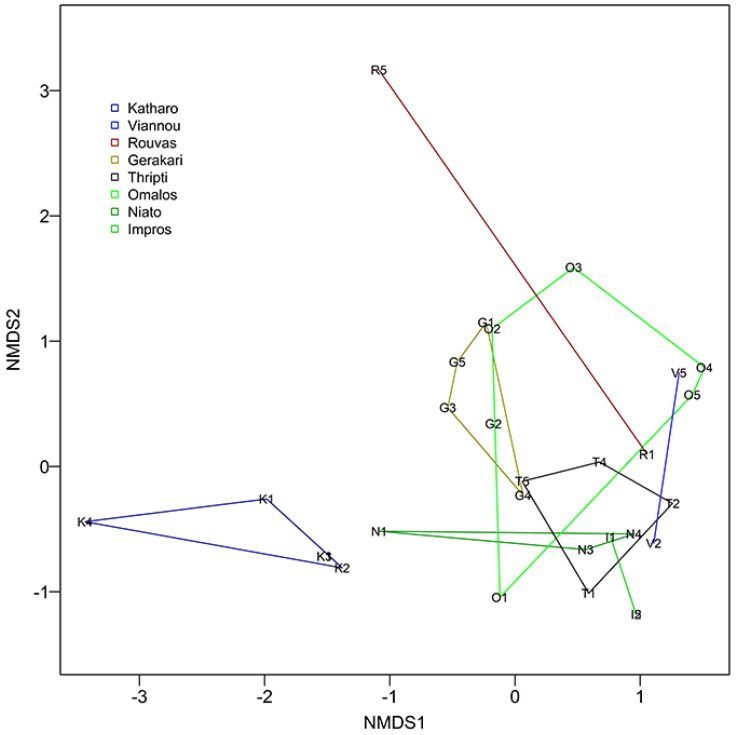
The centroids determined from the NMDS analysis showing the numerical diversity among the samples for each study site for Acari. The center of each centroid is indicated by a lettered square representing the study site (i.e., O: Omalos, N: Niato, I: Impros, G: Gerakari, R: Rouvas, V: Viannou, K: Katharo and T: Thripti).

More analyses were conducted for the 10 most numerous species of Acari (Table 2, Figure 4). It turned out that their occurrence was not uniform in either all of the study sites or all of the samples, as shown by their frequencies. The highest frequency was recorded for *Camisia horrida* (Hermann, 1804) (F: 32.5%), even though it was not an exceptionally abundant species. In turn, the species represented by high numbers of specimens, e.g., *E. tuberosus*, showed low frequencies (F: 10%) (Table 2).

The NMDS analysis (stress 0,08 for three dimensions) showed that the species composition and the population size determined the character of a given Acari assemblage and these two factors varied depending on the study site (Figure 4). In the sites of Rouvas and Viannou, oribatid mites were identified only in two samples. A similar result was obtained for the site of Impros, although here mites were detected in four samples, with three of them being identical and having the same coordinates. For this reason, in Figure 4, these sites are presented in the form of a straight line (as the data are arranged along a line) rather than a space. In this plot, the two samples from Rouvas are very distant from the first and second axis, which means that the species composition of these sites is completely different (there are no common species). A similarly large scatter of samples was observed for Niato (N1—differs considerably from the two other samples), Omalos (O1– considerable differences in the species composition from O3) and in Katharo, where K4 differs from K2, K3 and K5. The largest and most compact space is marked by samples from Thripti and Gerakari. The samples located close to each other show the similarity of the species assemblages of Acari. Thus, the Acari species found in the Omalos site O2 are most often found also in sample G1 from Gerakari. Similarly, the samples N3, N4 and V2 are close together in the NMDS plot, indicating that these share similar mite assemblages. In turn, Katharo shows an assemblage that is quite different than any other sites, with one sample, K4, being relatively distant from the other samples collected at site 3, and K5 had a similar species composition with N1 both in the axis systems MDS1 and MDS2 and MDS1 and MDS3. It is worth paying attention to the G1 and O2 samples, located close to each other in all dimensions, whose species composition is very similar. The sampling sites show rather diverse species assemblages as indicated by the limited number of overlapping segments and the high diversity within.

The conducted multilevel pattern analysis showed that Gerakari distinguishes itself by the presence of three species: *Eremaeus tuberosus* Gordeeva, 1970 (*p* = 0.0080), *Sphodrocepheus tridactylus* Woolley et Higgins, 1963 (*p* = 0.0370) and *Scheloribates pallidulus* (C. L. Koch, 1841) (*p* = 0.0387). In turn, Impros distinguishes itself by the occurrence of *Camisia horrida* (Hermann, 1804) (*p* = 0.0447), while for Rouvas it is *Platyliodes scaliger* (C. L. Koch, 1840) (*p* = 0.0467). For Gerakari and Omalos, it is the occurrence of *Ceratoppia quadridentata* (Haller, 1882) (*p* = 0.032), whereas Gerakari and Niato are characterized by the presence of *Zygoribatula exilis* (Nicolet, 1855) (*p* = 0.013).

**Figure 5:**
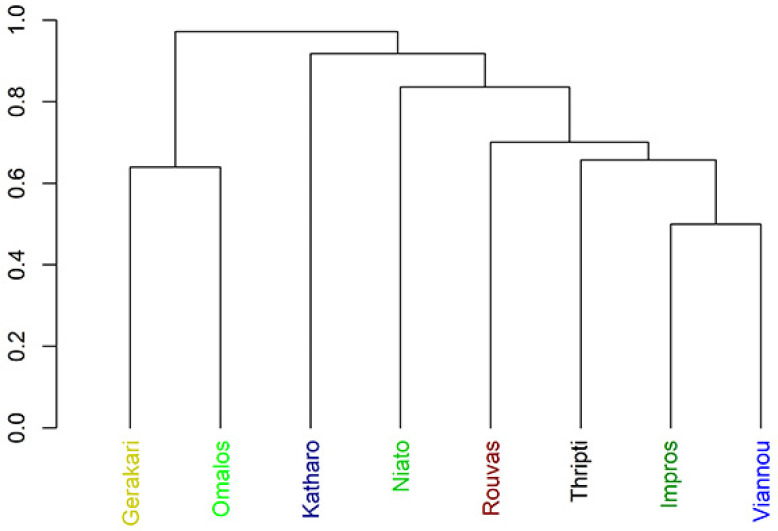
A cluster analysis showing the similarity of study sites depending on their Acari communities.

**Figure 6:**
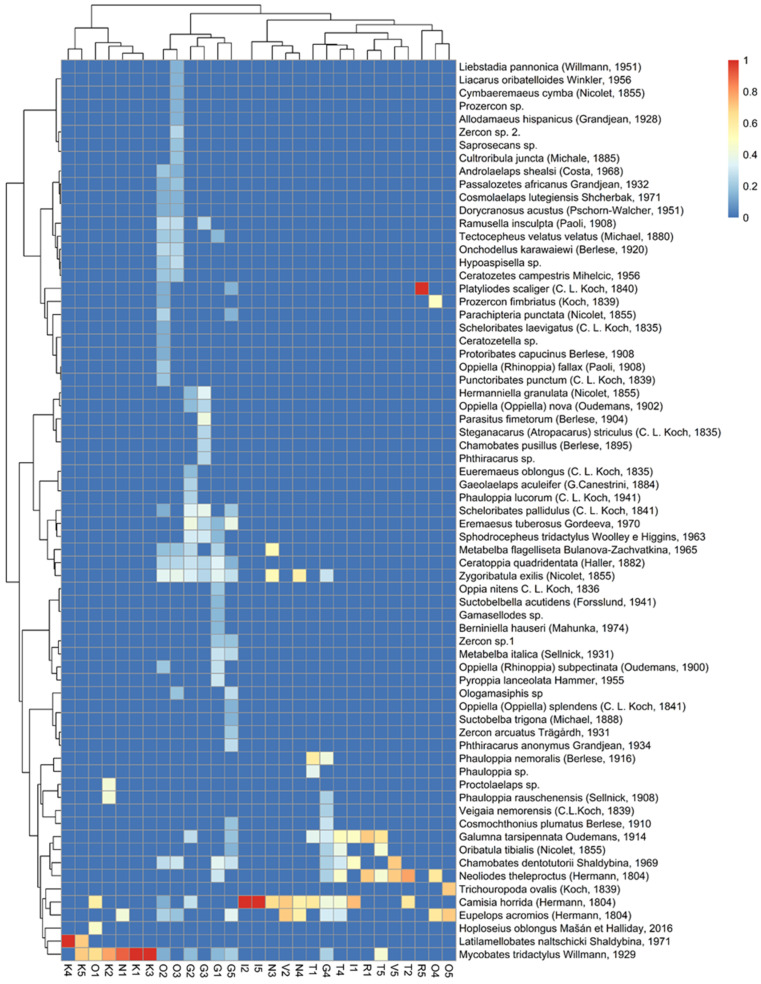
Heatmap showing species frequency.

The cluster analysis shows that the similarities between individual sites do not reflect their geographic proximity. Indeed, study sites within the same mountain range were considerably distant, e.g., Omalos, Niato and Impros are situated in the Levka Ori Mts, but Viannou and Katharo were in the same clusters (Figure 5). Because Katharo was very far from other locations in Figure 4 relative to the NMDS1 and in the cluster analysis, this location was at the end of the chain, the distinctiveness of this location was confirmed. The heatmap and chance of species co-occurrence is presented in Figure 6 and Table 3. The heatmap shows that in all samples from Katharo, one species was abundantly present (also found in other locations)—*Mycobates tridactylus* Willmann, 1929—and in two of these samples (K4 and K5), a second species, *Latilamellobates naltschicki* Shaldybina, 1971, was also abundant. It is important to note that this species was recorded only in these locations, which is why the NMDS analysis indicated the distinctiveness of the samples collected in Katharo, and the cluster analysis suggested cutting the groups at a distance level of 0.9 (Katharo forms a separate group). The species co-occurrence analysis revealed eight pairs of species for which there was a significant probability of their co-occurrence, exceeding the expected probability of their co-occurrence (Table 3). 

## 4. Discussion

The high number of arachnid taxa found in this study demonstrates the rich diversity of arthropods on Crete. The numerous publications describing species new to science [3,4,7,51], as well as the findings during this study of several taxa that are possibly new to science, show how much is still to be discovered concerning arthropods on Greek islands. In the analyzed material, at least one reported Acari species is probably new to science, e.g., *Hypoaspisella* sp. (which will be the subject of a separate taxonomic analysis). We would like to highlight the presence of several rare species, as well as species previously known outside the Mediterranean area, such as *Hoploseius oblongus* Mašán et Halliday, 2016, from Slovakia and Poland [52], which was recently found in France, Denmark, and Ukraine, and is also widely spread across Russia (from the Caucasus to the Arctic) [53]. Additional rare species include *Androlaelaps shealsi* (Costa, 1968), which was previously reported from Israel and Iran [54], and *Cosmolaelaps lutegiensis* Shcherbak, 1971, which has so far been recorded in Ukraine and Kazakhstan [55]. The above-mentioned species were identified in southern Europe for the first time, which highlights the importance of relict tree species such as *Z. abelicea* for providing microhabitats to non-typical Mediterranean arthropod species living in a Mediterranean context.

The microhabitats offered by *Z. abelicea* have been poorly studied up to the present, although considerable effort has been made to amend the situation in recent years. To date, only two studies concerning Acari have been conducted on *Z. abelicea* trees [5,7], while several studies have focused on Collembola [56,57] and one study investigated epiphytic bryophytes and lichens [18].

Our results (Figure 4 and Figure 5) show the high diversity of the arachnid communities at each study site. Even communities in sites located relatively close to one another within one mountain range, such as Impros and Niato, differed in terms of the character of the arachnid communities. Arachnid diversity can be influenced by a number of factors [58].These differences may be linked to differences in plant species composition and forest structure [1,59,60,61,62], including *Z. abelicea* tree size [18], or be due to differences in both current and past land-use and management, including grazing history [63,64]. However, most probably these differences highlight the high diversity of the microhabitats offered by *Z. abelicea* and highlight the isolation and fragmentation of the *Z. abelicea* stands and the microhabitats they offer, with no evident connections between stands.

The fact that Omalos and Gerakari showed a higher diversity than the other sites cannot be solely imputed to tree size (non-dwarfed) or land-use patterns (wooded pasture lands), as other sites show similar features. Gerakari was found to have a particularly rich bryophyte diversity and specific community composition [18], which may possibly also offer a higher diversity of microhabitats and therefore promote arachnid diversity. However, our results open the road to further investigations to address the strong differences in community composition and species diversity shown in our study. The aim of this study was not to define the abiotic and biotic factors determining the structure of mite communities. This will be the subject of further research, which will analyze, among other factors, thermal and humidity conditions, and the feeding base (e.g., fungi).

Acarological literature concerning Greece, including Crete, is relatively extensive. For example, Swirski and Ragusa [6] conducted acarological studies in Crete and reported several species from the family Phytoseiidae. However, a particularly important role is played by publications presenting descriptions of species new to science, since such results confirm the unique character of this island. Examples of such publications include the studies of Mahunka [3], who described *Dissorhina cretensis* belonging to the order Oribatida, Ujvári [4], who described *Prozercon rekaae* and *Zercon cretensis*, Stathakis and Papadoulis [51], who described *Typhlodromus* (*Anthoseius*) *creticus* and Witaliński and Gwiazdowicz [7], who described *Ologamasiphis zelkovae* from the order Mesostigmata.

For several decades, acarological studies have been conducted by collecting material from tree bark and determining the mite communities present in this microhabitat [65]. Research concerning the acarofauna of *Z. abelicea* trees is in line with this trend in acarology. Eight isolated populations of these trees are growing in the mountainous regions of Crete that are located at high altitudes. Some trees are magnificent and tall, with large trunk diameters, while others are stunted, with a dwarf habit and they are browsed by goats. As a result, the trunks of these trees, as well as the branches or twigs, are covered by diverse species of mosses and lichens [18]. In view of the above-mentioned environmental factors, it was assumed that the communities of arachnids, particularly mites, would differ at each research site.

The recorded results confirmed the proposed research hypothesis. The statistical analyses concerning all the communities of mites showed that each locality of *Z. abelicea* is inhabited by diverse communities. This is convincing, as it was shown both by the NMDS and cluster analysis (Figure 4 and Figure 5), which was significantly affected by the number and species composition of Acari. The largest numbers of Acari and the highest number of species were recorded for the Omalos and Gerakari sites.

## 5. Conclusions

In conclusion, it may be stated that the diversity of the arachnids found on *Z. abelicea* shows the availability of a wide variety of microhabitats. The low relatedness between the arachnid communities present in some neighboring sites shows the absence of interconnectivity between the *Z. abelicea* stands and their microhabitats and highlights the isolation and strong fragmentation of the forest stands on Crete. In the collected material, the most numerously represented orders were Oribatida (1056 individuals, 51 species) and Mesostigmata (78 individuals, 18 species). The most numerous species included *Zygoribatula exilis* (284 individuals) and *Eremaeus tuberosus* (210). Among other things, based on the results of the NMDS and cluster analyses, the arachnid communities recorded in Omalos and Gerakari were the most distinctive, as a result of the good microhabitat conditions, i.e., the presence of large, old trees. Moreover, our findings included relatively rare species, as well as a species that is new to science from the genus *Hypoaspisella*, which confirms the unique character of the mite communities colonizing the endemic *Zelkova abelicea* trees. The results of this pilot study justify the need to continue research on the microarthropods inhabiting this exceptional endemic tree. From a conservation perspective, assessing arachnid species richness and community composition, alongside other taxa, can help to orientate the decision around selecting priority areas for conservation [66].

## Figures and Tables

**Table 1:** Number of specimens (species) for each sample at every study site. Total number of specimens and species, standard error (SE), minimum and maximum per site are also given as well as Simpson’s index and Pielou’s Index.

Mountain		Levka Ori			Kedros	Psiloritis	Dikti		Thripti
Site		Omalos	Niato	Impros	Gerakari	Rouvas	Viannou	Katharo	Thripti
Sample	1 Ind (sp)	7 (4)	24 (2)	9 (4)	150 (19)	3 (3)	1 (1)	1 (1)	13 (4)
	2	186 (29)	0 (0)	1 (1)	134 (16)	1 (1)	2 (2)	11 (4)	13 (3)
	3	209 (27)	4 (3)	0 (0)	25 (14)	0 (0)	1 (1)	3 (1)	0 (0)
	4	5 (3)	3 (3)	1 (1)	41 (15)	1 (1)	0 (0)	2 (1)	18 (7)
	5	2 (2)	0 (0)	1 (1)	296 (20)	4 (4)	6 (6)	2 (2)	6 (4)
Individuals	Total	409	31	12	646	9	10	19	50
	Mean ± SE	81.8 ± 47.38	6.2 ± 4.52	2.4 ± 1.66	129.2 ± 48.43	1.8 ± 0.73	2.0 ± 1.05	3.8 ± 1.83	10.0 ± 3.15
	Min	2	0	0	25	0	0	1	0
	Max	209	24	9	296	4	6	11	18
Species	Total	43	5	4	50	7	9	5	10
	Mean	13.0 ± 6.14	1.6 ± 0.68	1.4 ± 0.68	16.8 ± 1.16	1.8 ± 0.73	2.0 ± 1.05	1.8 ± 0.58	3.6 ± 1.12
	Min	2	0	0	14	0	0	1	0
	Max	29	3	4	20	4	6	4	7
Simpson’s Index	Mean ± SE	0.64 ± 0.04	0.46 ± 0.19	0.17 ± 0.17	0.81 ± 0.04	0.35 ± 0.21	0.33 ± 0.20	0.19 ± 0.12	0.67 ± 0.04
	Min	0.50	0.08	0	0.67	0	0	0	0.62
	Max	0.72	0.67	0.67	0.88	0.75	0.83	0.50	0.79
Pielou’s Index	Mean ± SE	0.81 ± 0.09	0.73 ± 0.24	0.88 ± NA	0.76 ± 0.06	1 ± 0	1 ± 0	0.82 ± 0.18	0.88 ± 0.20
	Min	0.56	0.25	0.88	0.56	1	1	0.64	0.81
	Max	1.00	1	0.88	0.90	1	1	1	0.92

**Table 2:** The analysis of the 10 most numerous Acari species. N: number of specimens, Min: minimum number of specimens in the samples, Max: maximum number of specimens in the samples, Mean ± standard error. F: frequency, namely the proportion of the samples in which the species occurs (%), I: Intensity, namely the ratio of the number of specimens of every species to the number of samples in which the species was found, R: Relative density, namely the ratio of the number of specimens of a given species to the number of all samples.

Name of Species	N	Min	Max	Mean (±SE)	F (%)	I	R
*Camisia horrida* (Hermann, 1804)	40	0	10	3.08 ± 0.44	32.5	3.08	1.00
*Ceratoppia quadridentata* (Haller, 1882)	46	0	23	7.67 ± 1.19	15	7.67	1.15
*Chamobates dentotutorii* Shaldybina, 1969	83	0	35	10.38 ± 1.32	20	10.38	2.08
*Eremaeus tuberosus* Gordeeva, 1970	210	0	154	52.50 ± 4.98	10	52.50	5.25
*Eupelops acromios* (Hermann, 1804)	81	0	64	8.10 ± 2.19	25	8.10	2.03
*Galumna tarsipennata* Oudemans, 1914	22	0	6	2.75 ± 0.41	20	2.75	0.55
*Mycobates tridactylus* Willmann, 1929	48	0	23	4.00 ± 0.91	30	4.00	1.20
*Neoliodes theleproctus* (Hermann, 1804)	26	0	11	3.25 ± 0.69	20	3.25	0.65
*Scheloribates pallidulus* (C. L. Koch, 1841)	27	0	19	6.75 ± 1.59	10	6.75	0.68
*Zygoribatula exilis* (Nicolet, 1855)	284	0-	123	31.56 ± 2.72	22.5	31.56	7.10

**Table 3:** Significant co-occurrence of species.

The Name of the First Species	The Name of the Second Species	Likelihood of Co-Occurrence Greater than Expected
*Ceratoppia quadridentata* (Haller, 1882)	*Chamobates dentotutorii* Shaldybina, 1969	0.0335
*Ceratoppia quadridentata* (Haller, 1882)	*Metabelba flagelliseta* Bulanova-Zachvatkina, 1965	0.0030
*Ceratoppia quadridentata* (Haller, 1882)	*Zygoribatula exilis* (Nicolet, 1855)	0.0002
*Chamobates dentotutorii* Shaldybina, 1969	*Zygoribatula exilis* (Nicolet, 1855)	0.0374
*Eremaesus tuberosus* Gordeeva, 1970	*Zygoribatula exilis* (Nicolet, 1855)	0.0053
*Galumna tarsipennata* Oudemans, 1914	*Oribatula tibialis* (Nicolet, 1855)	0.0030
*Metabelba flagelliseta* Bulanova-Zachvatkina, 1965	*Zygoribatula exilis* (Nicolet, 1855)	0.0011
*Scheloribates pallidulus* (C. L. Koch, 1841)	*Zygoribatula exilis* (Nicolet, 1855)	0.0053

## Data Availability

The data presented in this study are available upon request from the corresponding author.

## Appendix A

**Table A1:** List of arachnida species in systematic order and dominance index (D).

Mountain	Levka Ori			Kedros	Psiloritis	Dikti		Thripti	
Taxon/Site	Omalos	Niato	Impros	Gerakari	Rouvas	Viannou	Katharo	Thripti	D
**ARANEAE**									
Amaurobiidae sp.				1					0.08
*Clubiona* sp. 1.	2								0.17
*Clubiona* sp. 2.							1		0.08
*Dipoena nigroreticulata* (Simon, 1880)	1								0.08
Dysderidae sp.				5					0.42
Gnaphosidae sp.				1					0.08
*Lathys stigmatisata* (Menge, 1869)					1	1			0.17
Linyphiidae sp.	5				2	2			0.76
Liocranidae sp.				2					0.17
*Pelecopsis elongata* (Wider, 1834)					2	1			0.25
Thomisidae sp.						1			0.08
*Xysticus* sp.				1	1				0.17
*Zodarion granulatum* Kulczyński, 1908				2					0.17
*Zodarion* sp.						1			0.08
**PSEUDOSCORPIONES**									
*Beierochelifer peloponnesiacus* (Beier, 1929)	1		4					7	1.01
*Neobisium* cf *validum* (L. Koch, 1873)	1			6					0.59
**ACARI, Mesostigmata**									
*Androlaelaps shealsi* Costa, 1968	4								0.34
*Cosmolaelaps lutegiensis* Shcherbak, 1971	2								0.17
*Gaeolaelaps aculeifer* (G. Canestrini, 1884)				3					0.25
*Gamasellodes* sp.				1					0.08
*Hoploseius oblongus* Mašán et Halliday, 2016	1								0.08
*Hypoaspisella* sp.	13								1.1
*Ologamasiphis* sp.	2			10					1.01
*Onchodellus karawaiewi* (Berlese, 1920)	15								1.26
*Parasitus fimetorum* (Berlese, 1904)				6					0.51
*Proctolaelaps* sp.							1		0.08
*Prozercon fimbriatus* (C.L. Koch, 1839)	2								0.17
*Prozercon rekaae* Ujvari, 2008	1								0.08
*Saprosecans* sp.	2								0.17
*Trichouropoda ovalis* (C.L. Koch, 1839)	1								0.08
*Veigaia nemorensis* (C.L. Koch, 1839)				1					0.08
*Zercon arcuatus* Trägårdh, 1931				3					0.25
*Zercon athiasi* Vincze, 1965	6								0.51
*Zercon* sp.				4					0.34
**ACARI, Oribatida**									
*Allodamaeus hispanicus* (Grandjean, 1928)	1								0.08
*Berniniella hauseri* (Mahunka, 1974)				1					0.08
*Camisia horrida* (Hermann, 1804)	3	3	6	16		1		11	3.37
*Ceratozetes campestris* Mihelčič, 1956	6								0.51
*Ceratoppia quadridentata* (Haller, 1882)	14			32					3.88
*Ceratozetella* sp.	1								0.08
*Chamobates dentotutorii* Shaldybina, 1969	29		1	51		1		1	7
*Chamobates pusillus* (Berlese, 1895)				1					0.08
*Cosmochthonius plumatus* Berlese, 1910				4					0.34
*Cultroribula juncta* (Michael, 1885)	2								0.17
*Cymbaeremaeus cymba* (Nicolet, 1855)	1								0.08
*Dorycranosus acustus* (Pschorn-Walcher, 1951)	2								0.17
*Eremaeus tuberosus* Gordeeva, 1970				210					17.71
*Eueremaeus oblongus* (C.L. Koch, 1835)				1					0.08
*Eupelops acromios* (Hermann, 1804)	10	2		67		1		1	6.83
*Galumna tarsipennata* Oudemans, 1914			1	10	1			10	1.85
*Hermanniella granulata* (Nicolet, 1855)				4					0.34
*Latilamellobates naltschicki* Shaldybina, 1971							3		0.25
*Liacarus oribatelloides* Winkler, 1956	1								0.08
*Liebstadia pannonica* (Willmann, 1951)	1								0.08
*Metabelba flagelliseta* Bulanova-Zachvatkina, 1965	4	1		8					1.1
*Metabelba italica* (Sellnick, 1931)				17					1.43
*Mycobates tridactylus* Willmann, 1929	4	23		7			13	1	4.05
*Neoliodes theleproctus* (Hermann, 1804)	2			12	1	1		10	2.19
*Oppia nitens* C.L. Koch, 1836				2					0.17
*Oppiella* (*Oppiella*) *nova* (Oudemans, 1902)				2					0.17
*Oppiella* (*Oppiella*) *splendens* (C.L. Koch, 1841)				1					0.08
*Oppiella* (*Rhinoppia*) *fallax* (Paoli, 1908)	4								0.34
*Oppiella* (*Rhinoppia*) *subpectinata* (Oudemans, 1900)	3			8					0.93
*Oribatula tibialis* (Nicolet, 1855)				2				3	0.42
*Parachipteria punctata* (Nicolet, 1855)	6			1					0.59
*Passalozetes africanus* Grandjean, 1932	3								0.25
*Phauloppia lucorum* (C.L. Koch, 1941)				3					0.25
*Phauloppia nemoralis* (Berlese, 1916)				10				5	1.26
*Phauloppia rauschenensis* (Sellnick, 1908)				1			1		0.17
*Phauloppia* sp.								1	0.08
*Phthiracarus anonymus* Grandjean, 1934				9					0.76
*Phthiracarus* sp.				1					0.08
*Platyliodes scaliger* (C.L. Koch, 1840)	1			1	1				0.25
*Protoribates capucinus* Berlese, 1908	1								0.08
*Punctoribates punctum* (C.L. Koch, 1839)	3								0.25
*Pyroppia lanceolata* Hammer, 1955				10					0.84
*Ramusella insculpta* (Paoli, 1908)	20			1					1.77
*Scheloribates laevigatus* (C.L. Koch, 1835)	1								0.08
*Scheloribates pallidulus* (C.L. Koch, 1841)	1			26					2.28
*Sphodrocepheus tridactylus* Woolley et Higgins, 1963				13					1.1
*Steganacarus* (*Atropacarus*) *striculus* (C.L. Koch, 1835)				1					0.08
*Suctobelba trigona* (Michael, 1888)				1					0.08
*Suctobelbella acutidens* (Forsslund, 1941)				1					0.08
*Tectocepheus velatus velatus* (Michael, 1880)	8			1					0.76
*Zygoribatula exilis* (Nicolet, 1855)	218	2		64					23.95

## References

[1] Samu F., Lengyel G., Szita E., Bidló A., Ódor P. (2014). The effect of forest stand characteristics on spider diversity and species composition in deciduous-coniferous mixed forests. J. Arachnol..

[2] Bosmans R., Van Keer J., Russell-Smith A., Kornestedt T., Alderweireldt M., Bosselaers J., De Konninck H. (2013). Spiders of Crete. A catalogue with all currently known species (Araneae) from the Greek island of Crete. Arachnological Contributions. Newsl. Bel. Arachnol. Soc..

[3] Mahunka S. (2008). *Dissorhina cretensis* n. sp. and some other remarkable oribatid mites (Acari: Oribatida) from Crete, Greece. Opusc. Zool. Budapest.

[4] Ujvári Z. (2008). Zerconid mites (Acari: Mesostigmata: Zerconidae) from Crete, Greece, with description of two new species. Opusc. Zool. Bp..

[5] Stathakis T.I. (2011). Predatory Mites of the Family Phytoseiidae (Acari: Mesostigmata) on Native Plants of Crete. Master’s Thesis.

[6] Swirski E., Ragusa S. (1976). Notes on predacious mites of Greece, with a description of five new species (Mesostigmata: Phytoseiidae). Phytoparasitica.

[7] Witaliński W., Gwiazdowicz D.J. (2023). Four new species of mites in *Ologamasiphis* and *Holzmannia* genera, and a new *Juvaria* subgenus defined (Parasitiformes: Parasitidae). Acarologia.

[8] Henderickx H. (2002). A new *Larca* (Arachnida: Pseudoscorpiones: Larcidae) from Crete. Bull. Brit. Arachnol. Soc..

[9] Deltshev C. (2011). The faunistic diversity of cave-dwelling spiders (Arachnida, Araneae) of Greece. Arachnol. Mitt..

[10] Grandcolas P., Nattier R., Trewick S. (2014). Relict species: A relict concept. Trends Ecol. Evol..

[11] Kozlowski G., Gratzfeld J. (2013). Zelkova—An Ancient Tree. Global Status and Conservation Action.

[12] Kozlowski G., Bétrisey S., Song Y., Fazan L., Garfì G. (2018). The Red List of Zelkova.

[13] Barbagallo S. (2002). *Zelkovaphis trinacriae*, a new Eriosomatine aphid genus and species living on Zelkova in Sicily (Rhynchota: Aphididae). Boll. Zool. Agr. Bach..

[14] Barbagallo S., Cocuzza G.E., Suma P. (2009). *Zelkovaphis trinacriae*, an Eriosomatine aphid relict living in Sicily on *Zelkova sicula*. Redia.

[15] Rejžek M., Svátek M., Šebesta J., Adolt R., Maděra P., Matula R. (2016). Loss of a single tree species will lead to an overall decline in plant diversity: Effect of *Dracaena cinnabari* Balf. f. on the vegetation of Socotra Island. Biol. Conserv..

[16] Gol A., Sadeghi-Namaghi H., de Lillo E. (2018). Two new species of eriophyoid mites (Acari: Trombidiformes: Eriophyoidea) on *Zelkova carpinifolia* (Ulmaceae) from Iran. Syst. Appl. Acarol..

[17] Maděra P., Habrová H., Šenfeldr M., Kholová I., Lvončík S., Ehrenbergerová L., Roth M., Nadezhdina N., Němec P., Rosenthal J. (2019). Growth dynamics of endemic *Dracaena cinnabari* Balf. of Socotra Island suggest essential elements for a conservation strategy. Biologia.

[18] Fazan L., Gwiazdowicz D.J., Fragnière Y., Fałtynowicz W., Ghosn D., Remoundou H., Rusińska A., Urbański P., Pasta S., Garfi G. (2022). Factors influencing the diversity and distribution of epiphytic lichens and bryophytes on the relict tree *Zelkova abelicea* (Lam.) Boiss. (Ulmaceae). Lichenologist.

[19] Gwiazdowicz D.J., Skarżyński D., Fazan L., Fragnière Y., Ghosn D., Kozlowski G., Kuźmiński R., Remoundou H., Zawieja B. (2022). Microarthropods Living on the Endemic Tree *Zelkova abelicea* (Ulmaceae) with Particular Attention to Collembola Diversity. Forests.

[20] Mai D.H. (1991). Palaeofloristic changes in Europe and the confirmation of the Arctotertiary-Palaeotropical geofloral concept. Rev. Palaeobot. Palynol..

[21] Fazan L., Stoffel M., Frey D.J., Pirintsos S., Kozlowski G. (2012). Small does not mean young: Age estimation of severely browsed trees in anthropogenic Mediterranean landscapes. Biol. Conserv..

[22] Hilszczański J. (2011). New data on the occurrence of Stephanids (Hymenoptera: Stephanidae) in Turkey and Greece. Opole Sci. Soc. Nat. J..

[23] Roberts M.J. (1985). The Spiders of Great Britain and Ireland.

[24] Almquist S. (2006). Swedish Araneae, part 2—Families Dictynidae to Salticidae. Insect Syst. Evol. Suppl..

[25] Nentwig W., Blick T., Bosmans R., Gloor D., Hänggi A., Kropf C. (2020). Spiders of Europe. Version 07.2020. https://www.araneae.nmbe.ch.

[26] Karg W. (1993). Acari (Acarina), Miben Parasitiformes (Anactinochaeta), Cohors Gamasina Leach. Raubmilben.

[27] Mašán P. (2001). Mites of the cohort Uropodina (Acarina, Mesostigmata) in Slovakia. Annot. Zool. Bot..

[28] Gwiazdowicz D.J. (2007). Ascid Mites (Acari, Mesostigmata) from Selected Forest Ecosystems and Microhabitats in Poland.

[29] Norton R.A., Dindal D.L. (1990). Acarina: Oribatida. Soil Biology Guide.

[30] Solhøy S.T., Smol J.P., Birks H.J.B., Last W.M. (2001). Oribatid mites. Tracking Environmental Changes in Lake Sedi-Ments.

[31] Olszanowski Z. (1996). A Monograph of the Nothridae and Camisiidae of Poland (Acari: Oribatida: Crotonioidea).

[32] Weigmann G. (2006). Hornmilben (Oribatida). Die Tierwelt Deutschlands und der angrenzenden Meeresteile, 76. Teil.

[33] Niedbała W. (2008). Ptyctimous Mites (Acari, Oribatida) of Poland.

[34] Simpson E.H. (1949). Measurement of diversity. Nature.

[35] Pielou E.C. (1966). The measurement of diversity in different types of biological collections. J. Theoret. Biol..

[36] Odum E.P. (1971). Fundamentals of Ecology.

[37] Patil V.V., Kulkarni H.V. (2012). Comparison of confidence intervals for the Poisson mean: Some new aspects. REVSTAT–Stat. J..

[38] Nagel L., Robb T., Forbes M.R. (2010). Inter-annual variation in prevalence and intensity of mite parasitism relates to appearance and expression of damselfly resistance. BMC Ecol..

[39] Clarke K.R., Warwick R.M. (1994). Change in Marine Communities: An Approach to Statistical Analysis and Interpretation.

[40] Bray J.R., Curtis J.T. (1957). An Ordination of the Upland Forest Communities of Southern Wisconsin. Ecol. Monog..

[41] Legendre P., Gallagher E.D. (2001). Ecologically meaningful transformations for ordination of species data. Oecologia.

[42] Dufrene M., Legendre P. (1997). Species assemblages and indicator species: The need for a flexible asymmetrical approach. Ecol. Monog..

[43] Hellinger E. (1909). Neue Begründung der Theorie quadratischer Formen von unendlich vielen Veränderlichen. J. Reine Angewand. Mathemat..

[44] Veech J.A. (2013). A Probabilistic Model for Analysing Species Co-Occurrence: Probabilistic Model. Glob. Ecol. Biogeogr..

[45] (2020). R Core Team R: A Language and Environment for Statistical Computing.

[46] Oksanen J., Blanchet F.G., Friendly M., Kindt R., Legendre P., Minchin P.R., O’Hara R.B., Simpson G.L., Solymos P., Stevens M.H.H. (2022). Vegan: Community Ecology Package.

[47] Raivo Kolde R. (2019). Pheatmap: Pretty Heatmaps. R Package Version. https://CRAN.R-project.org/package=pheatmap.

[48] Griffith D.M., Veech J.A., Marsh C.J. (2016). Cooccur: Probabilistic species co-occurrence analysis in R. J. Stat. Softw..

[49] De Cáceres M., Legendre P. (2009). Associations between species and groups of sites: Indices and statistical inference. Ecology.

[50] Wickham H. (2016). Ggplot2: Elegant Graphics for Data Analysis.

[51] Stathakis T.I., Papadoulis G.T. (2012). New records of phytoseiid mites from Greece with description of *Typhlodromus* (*Anthoseius*) *creticus* sp. nov. (Acari: Phytoseiidae). Int. J. Acarol..

[52] Gdula A.K., Skubała P., Zawieja B., Gwiazdowicz D.J. (2021). Mite communities (Acari: Mesostigmata, Oribatida) in the red belt conk, *Fomitopsis pinicola* (Polyporales), in Polish forests. Exp. Appl. Acarol..

[53] Andrianov B.V., Makarova O.L., Goryacheva I.I., Zuev A.G. (2022). The Range, Transmitting Insects, and Mitochondrial DNA Polymorphism of Gamasid Mite *Hoploseius oblongus* (Mesostigmata, Blattisociidae), Obligate Mycobiont on Bracket Fungus *Fomitopsis pinicola* (Polyporales, Basidiomycota). Rus. J. Gen..

[54] Ebrahimi N., Noei J. (2022). Checklist of mites associated with stored products (Arachnida: Acari) of Iran. Persian J. Acarol..

[55] Joharchi O., Issakova A.K., Asyamova O.S., Sarcheshmeh M.A., Tolstikov A.V. (2020). Some soil-inhabiting mites (Acari: Mesostigmata) from Kazakhstan, with description of a new species of *Gaeolaelaps* Evans & Till (Acari: Laelapidae). Zootaxa.

[56] Skarżyński D., Gwiazdowicz D.J. (2022). A redescription of *Hypogastrura gisini* Strenzke, 1954 (Collembola: Hypogastruridae) and description of a new related species from Crete (Greece). Zootaxa.

[57] Smolis A., Skarżyński D., Gwiazdowicz D.J. (2023). New species of Neanuridae (Collembola) living on the endemic tree *Zelkova abelicea* in Crete. Zootaxa.

[58] Larrivee M., Buddle C.M. (2010). Scale dependence of tree trunk spider diversity patterns in vertical and horizontal space. Ecoscience.

[59] Pearce J.L., Venier L.A., Eccles G., Pedlar J., McKenney D. (2004). Influence of habitat and microhabitat on epigeal spider (Araneae) assemblages in four stand types. Biodiv. Conserv..

[60] Schuldt A., Fahrenholz N., Brauns M., Migge-Kleian S., Platner C., Schaefer M. (2008). Communities of ground-living spiders in deciduous forests: Does tree species diversity matter?. Biodiv. Conserv..

[61] Schuldt A., Bruelheide H., Hardtle W., Assmann T. (2012). Predator assemblage structure and temporal variability of species richness and abundance in forests of high tree diversity. Biotropica.

[62] Korenko S., Kula E., Simon V., Michalkova V., Pekár S. (2011). Are arboreal spiders associated with particular tree canopies?. N.-West. J. Zool..

[63] Paschetta M., La Morgia V., Masante D., Negro M., Rolando A., Isaia M. (2013). Grazing history influences biodiversity: A case study on ground-dwelling arachnids (Arachnida: Araneae, Opiliones) in the Natural Park of Alpi Marittime (NW Italy). J. Ins. Conserv..

[64] Boieiro M., Matthews T.J., Rego C., Crespo L., Aguiar C.A.S., Cardoso P., Rigal F., Silva I., Pereira F., Borges P.A.V. (2018). A comparative analysis of terrestrial arthropod assemblages from a relict forest unveils historical extinctions and colonization differences between two oceanic islands. PLoS ONE.

[65] Kiełczewski B., Seniczak S. (1971). Mechowce (Oribatei) występujące na świerku pospolitym. Pr. Kom. Nauk Roln. Kom. Nauk Leśn. PTPN.

[66] Bondoso Cardoso P.M. (2004). The Use of Arachnids (Class Arachnida) in Biodiversity Evaluation and Monitoring of Natural Areas. Ph.D. Thesis.

